# The *foraging* Gene Is Involved in the Presence of Wings and Explorative Behaviours in Parthenogenetic Females of the Aphid *Myzus persicae*

**DOI:** 10.3390/life12030369

**Published:** 2022-03-03

**Authors:** Mauro Mandrioli, Gian Carlo Manicardi

**Affiliations:** 1 Department of Life Sciences, University of Modena and Reggio Emilia, Biology Building, Via Campi 213/D, 41125 Modena, Italy; 2 Department of Life Sciences, University of Modena and Reggio Emilia, Padiglione Besta, Via Amendola 2, 41122 Reggio Emilia, Italy; manigi04@unimore.it

**Keywords:** foraging, behavioural plasticity, winged aphids, peach potato aphid

## Abstract

The *foraging* gene (*for*) encodes for a cyclic guanosine monophosphate (cGMP)-dependent protein kinase involved in behavioural plasticity in aphids and in other insects. In this paper, we analysed the complete *for* sequence in eight clones of the peach potato aphid *Myzus persicae,* reporting the presence of nonsense and frameshift mutations in three studied clones characterized by a reduced number of winged females and by the absence of exploratory behaviours. Quantitative PCR experiments evidenced similar results in clones possessing *for* genes with a conserved coding sequence, but low expression levels. The comparison of the *for* transcriptional level in *Myzus persicae persicae* and *Myzus persicae nicotianae* showed very different expression in the two studied *M. p. nicotianae* clones so that our data did not support a previous hypothesis suggesting that a differential *for* expression was related to ecological specialization of *M. p. nicotianae*. In view of its role in both the dispersal of winged females and exploratory behaviours, the screening of the *for* sequences could be useful for predicting invasions of cultivated areas by peach potato aphids.

## 1. Introduction

In the last decades, several well-studied examples that shed light on genes and pathways underlying insect behavioural plasticity have been described [1,2,3,4,5]. In particular, single genes that affect the regulation of complex, yet distinct, behaviour patterns have been identified, suggesting that mutations altering their expression may vary the response to specific environmental stimuli generating behavioural plasticity [1,3,4,5,6].

Several recent achievements in this research field have been related to the *foraging* gene (*for*), which encodes a cyclic guanosine monophosphate (cGMP)-dependent protein kinase (PKG), suggesting that it may regulate different behaviours in several insects, including Diptera, Hymenoptera, Coleoptera and Hemiptera [1,3,5,6,7,8,9,10].

Originally identified as a behaviourally polymorphic gene that drives alternative foraging strategies in the fruit fly *Drosophila melanogaster* [11], the *for* gene has been successively deeply studied in social insects, where it has been observed that its differential expression plays a key role in the division of labour in diverse castes [5,6,12]. In particular, *for* regulates worker transitions between behavioural tasks and specific behavioural traits associated with morphological castes [5].

Although the specific neurological role of *for* in the insect brain is currently unknown, studies in some insect species indicated that PKG signalling plays a conserved role in the neuronal plasticity of sensory, cognitive and motor functions, which underlie behaviours related to appetitive learning, aggression, stress response, phototaxis and response to pheromones [5].

A *for* gene orthologue has also been identified in the pea aphid *Acyrthosiphon pisum* (*Apfor*) [13,14], where it is highly expressed in adults reared under crowded conditions. In particular, it has been suggested that *Apfor* may trigger the shift from sedentary to exploratory behaviour [13].

A *for* orthologue has also been studied in the peach potato aphid *Myzus persicae* (*Mpfor*) comparing generalist biotypes with tobacco-specialized races (generally identified as *Myzus persicae nicotianae*), suggesting that *Mpfor* influences the process of host searching and the evolution of ecological specialization [14]. At the same time, it has been suggested that low levels of *for* expression are related to a reduced PKG expression, resulting in reduced mobility, foraging activity and dispersal [14].

Interestingly, Tapia and colleagues [14] suggested that the low *for* expression observed in *M. p. nicotianae* could reinforce differences in diet breadth and ecological specialization by, for instance, reducing resource-searching behaviours and mobility. This hypothesis has been supported by the presence of a higher *for* transcription in alates that are responsible for host searching and dispersal in generalist clones in respect to specialized ones [14].

Considering that the dispersal of winged females is a key element to determine invasiveness of aphids, here, we compared the *for* sequence in eight *M. persicae* clones in order to evaluate if the screening of the *for* sequence could be useful to predict invasions of cultivated areas by peach potato aphids. Lastly, we compared the *for* expression in *M. p. persicae* and *M. p. nicotianae* to confirm the role of *Mpfor* in promoting ecological specialization.

## 2. Materials and Methods

### 2.1. Sampling and Rearing

Specimens of *M. persicae* were obtained from eight aphid lineages maintained as a colony of parthenogenetic females on pea *Pisum sativum* (cv ‘Meraviglia d’Italia’) plants at 19 °C with a light–dark regime of 16 h light and 8 h darkness that ensures parthenogenic reproduction (virginiparous females). Aphids were transferred to fresh pea plants once a week.

Aphids were reared in parallel at low and high population densities. In particular, five wingless parthenogenic females were transferred in an insect box and their progenies collected after a week. This low population density condition provided good food quality and a large space for female reproduction. High population density was obtained by transferring thirty females in a box containing a single pea plant and left there, giving rise to several generations until, after a week, crowded conditions were achieved.

Aphid clones were collected from three countries: *M. persicae* clone 1GK was collected from Greece and kindly supplied by John Margaritopoulos (Greece); clones K1, K10, D type and 229 were collected from Scotland and were kindly supplied by Brian Fenton (Scotland); clones 1ITA, 33H and 7 were collected from Italy and were kindly supplied by Emanuele Mazzoni (Italy). Clones 1GK and K1 were previously identified as *M. p. nicotianae* using microsatellite-based and chromosomal analyses [15,16]. A detailed description of each clone is reported in Table 1.

Isofemale clonal lineages were obtained from lines of aphids issuing from a single female and maintained in culture in the laboratory on pea plants in order to obtain homogeneous aphid samples. Clonal lines are periodically checked with different biochemical, molecular and biological assays in order to ensure the stability of the biological traits (chromosomal number, resistance type and level, reproductive modalities, etc.) observed at their first identification.

Aphids reared in high population densities have also been used to evaluate the presence of winged adults and wingless foragers. In particular, the number of both winged adults and unwinged walking aphids exploring the insect box was evaluated after a week of breeding in crowded conditions. This approach evidenced an abundant presence of both alate females and walking foragers in response to crowding clones 1ITA, 1 GK, K10, D-type and 229, whereas clones 33H, K1 and 7 consisted of unwinged females only, even if maintained on crowded pea plants.

### 2.2. RNA Extraction and foraging Gene Amplification

RNA extraction was performed with the *SV Total RNA Isolation System* (Promega), according to the supplier’s suggestions. RNA samples were quantified by spectrophotometric absorbance measurements using a NanoDrop™ ND1000 Spectrophotometer (Thermo Fisher Scientific, Waltham, MA, USA).

Amplification of an internal 807 bp-long portion of the *for* gene was performed by RT-PCR with the *Access RT-PCR System* (Promega) and the primers F-for (5′-GAGACGTTCTACAATGCTGGA) and R-for (5′-AGCAAAACCAAAGTCGACCA) at an annealing temperature of 57 °C for 1 min using 0.5 µg of polyA+ mRNA. Primers were designed using the webtool *Primer3* (freely available at the address https://primer3.ut.ee/, last access 28 January 2022) using the template sequence JF776573.1, annotated as *for* orthologue in the pea aphid *A. pisum*. RACE amplification was carried out to complete the *for* sequence using the *5′/3′ RACE Kit* (Roche), according to the supplier’s instructions.

Amplified fragments were cloned with the *TA Cloning^®^ kit* (Invitrogen) and transformed into electrocompetent *One shot^®^ TOP10* *Escherichia coli* cells (Invitrogen). Recombinant plasmids from ten positive colonies for each aphid clone were extracted, purified and sequenced at *BMR Genomics* (Padua, Italy).

Sequence alignments were performed using the software *Biogen^®^ CLC sequence viewer* (Aarhus, Denmark) and with the nBLAST and pBLAST tools available at NCBI (http://blast.ncbi.nlm.nih.gov/Blast.cgi, last access 28 January 2022). The analysis of the conserved domains in the coded foraging proteins was carried out using the *Conserved Domain Search* tool available at NCBI (https://www.ncbi.nlm.nih.gov/Structure/cdd/wrpsb.cgi, last access 28 January 2022).

### 2.3. qPCR Experiments and Statistical Analysis

The comparison of the expression level of the *for* genes in the eight *M. persicae* clones was accomplished via quantitative real-time PCR experiments (qPCR). In particular, RNA samples for qPCR were extracted from a pool of ten *M. persicae* wingless parthenogenic adults collected after a week of breeding in crowded conditions using the *Quick-RNA™ Miniprep Kit* (Zymo Research, Freiburg, Germany) following the manufacturer’s protocol. All RNA samples were double-checked for purity and quantified using both Qubit^®^ RNA HS Assays (Invitrogen, Carlsbad, CA, USA) and NanoDrop™ ND1000 Spectrophotometer (Thermo Fisher Scientific, Waltham, MA, USA).

The mRNA was reverse-transcribed to cDNA using the *iScript^®^ cDNA Synthesis Kit* (Bio-Rad Laboratories, Inc., Hercules, CA, USA) according to the manufacturer’s instructions. The qPCR reaction was performed using a *SsoAdvanced™ Universal SYBR^®^ Green Supermix* (Bio-Rad Laboratories, Inc., Hercules, CA, USA) with the specifically designed primers F-for_qPCR (5′-TGGAGTCTTGTCTTGGTGGT) and R-for_qPCR (5′-ATGCTTCAAGACACATCCG), following instructions provided by the manufacturer and using 1 µg of polyA+ mRNAs.

The applied thermal profile was as follows: 95 °C for 10 min (1 cycle), 95 °C for 2 min, and 58 °C for 30 s (30 cycles). All the reactions were performed in triplicate on a *CFX96 Touch Real-time PCR detection system* (Bio-Rad Laboratories, Inc., Hercules, CA, USA). Once the amplification reaction was completed, the melting curves were inspected for all the amplicons. Relative quantification of qPCR data was obtained through the 2^−(ΔΔCt)^ method, according to Livak and Schmittgen [17]. Statistical analyses were performed with SPSS Statistics 25 (IBM Corp, released 2017, Armonk, NY, USA). One-way analysis of variance (ANOVA) was applied (Levene test, *p* < 0.05).

## 3. Results and Discussion

The combined use of RT-PCR and RACE allowed the amplification of the complete *for* gene in the eight studied *M. persicae* clones, showing the presence of a 2310 bp long coding sequence for all *M. persicae* clones. The length of the *Mpfor* coding sequence is in agreement with data reported in the pea aphid *A. pisum*, where two orthologues have been identified with respective sizes of 2331 bp for *Apfor1* and 2112 bp for *Apfor2* [13].

As reported by Tares et al. [13], the *Apfor* orthologues differ in the first two exons, whereas they perfectly overlap in their following sequence. In particular, exons 1 and 2 of *Apfor2* are located within the intron 2 of the *Apfor1* gene suggesting that these exons are spliced in the *Apfor1* variant. The presence of a single *for* gene in *M. persicae* supports the occurrence of a specific duplication in the *A. pisum* lineage.

The *M. persicae* coded foraging protein showed a 99% and 96% identity with the *A. pisum* Apfor2 and the *D. melanogaster* foraging respectively, whereas a lower identity (86.7%) was obtained for the Apfor1 protein, suggesting that this probably represents a paralogous copy of the *for* gene.

**Figure 1:**
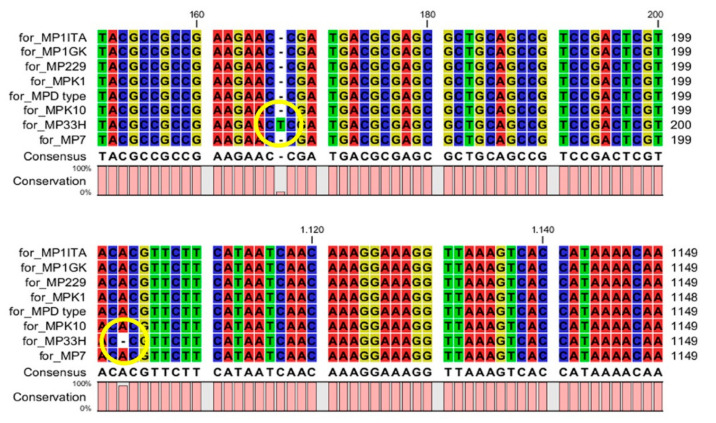
Nucleic sequence alignment appeared in the *M. persicae* clone 33H two frameshift mutations (shown by the yellow circles) occurring, respectively, in portion 150–200 and 1100–1149 of the *Mpfor* gene.

**Figure 2:**
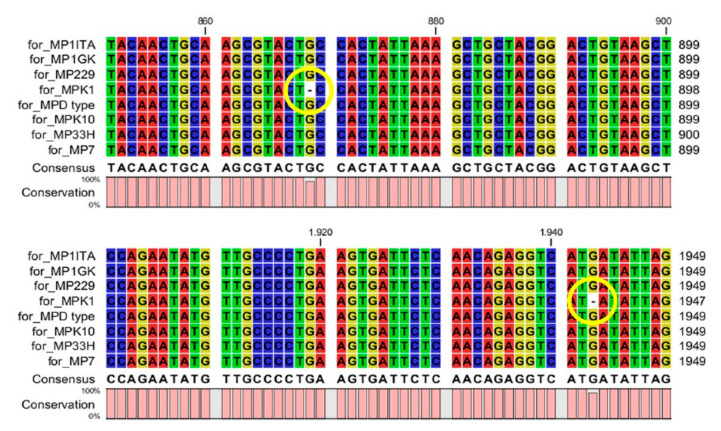
Nucleic sequence alignment evidenced in the *M. persicae* clone K1 two frameshift mutations (shown by the yellow circles) occurring, respectively, in portion 850–899 and 1900–1949 of the *Mpfor* gene.

**Figure 3:**
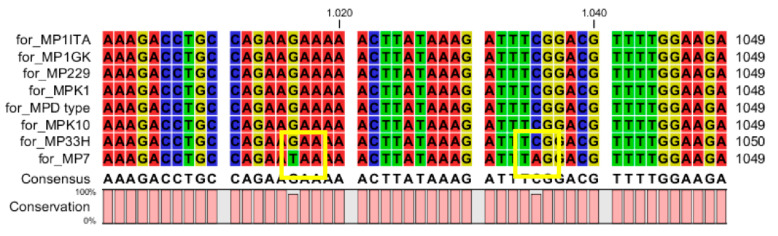
Nucleic sequence alignment evidenced in the *M. persicae* clone 7 two nonsense mutations (shown by the yellow squares) occurring in the portion 1000–1049 of the *Mpfor* gene. In particular, in the first mutation the GAA codon-coding for serine is changed into the TAA codon corresponding to a stop codon. Similarly, in the second mutation the TCG codon coding for glutamic acid is changed into the TAG codon corresponding to a stop codon.

Nucleic sequence alignment evidenced a very high sequence conservation in the *M. persicae* clones 1ITA, 1GK, 229, K10 and D-type, with sequence identity ranging from 99.69 to 100% (Table 2). Interestingly, despite the high conservation of their DNA sequence in terms of whole sequence identity, clones 33H, K1 and 7 possessed severe mutations affecting the conservation of the coded foraging peptide (Figure 1, Figure 2 and Figure 3). In particular, frameshift mutations were present in clones 33H (Figure 1) and K1 (Figure 2), whereas nonsense mutations were observed in clone 7 (Figure 3).

**Figure 4:**
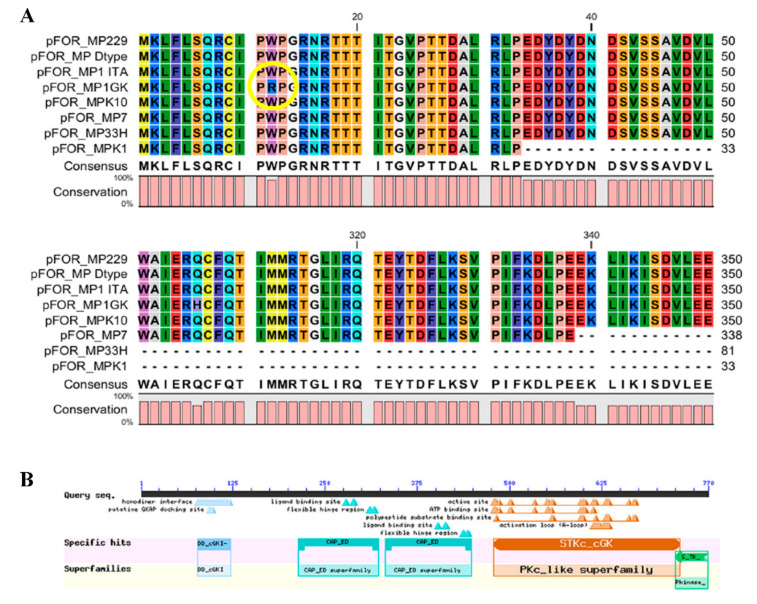
Protein sequence alignment evidenced in the *M. persicae* clone 1GK a missense mutation (**A**) (shown by the yellow circle), but the coded protein possessed both the conserved domains and active sites typically present in functional foraging proteins (**B**). Conserved domains are visualized in the line labelled “Specific Hits”, whereas “Superfamilies” represent the cluster of evolutionary conserved domains identified in the coded foraging proteins (labelled as “Query Seq”).

**Figure 5:**
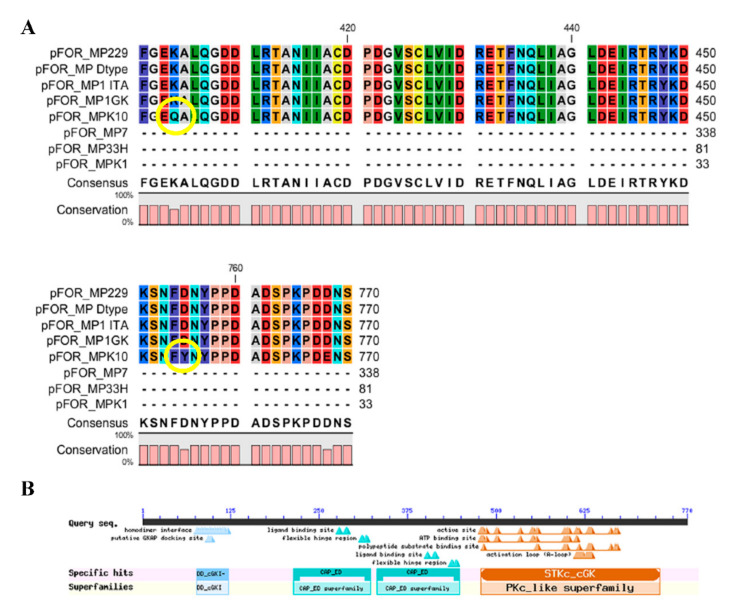
Protein sequence alignment evidenced in the *M. persicae* clone K10 two missense mutations (**A**) (shown by the yellow circles), but the coded protein possessed both conserved domains and actives sites typically present in functional foraging proteins (**B**). Conserved domains are visualized in the line labelled “Specific Hits”, whereas “Superfamilies” represent the cluster of evolutionary conserved domains identified in the coded foraging proteins (labelled as “Query Seq”).

The analysis of the foraging coded peptide evidenced the presence of missense mutations in clones 1GK (Figure 4) and K10 (Figure 5), but they did not change the structure of both functional domains and active sites suggesting that these mutations did not alter the functionality of the coded protein.

**Figure 6:**
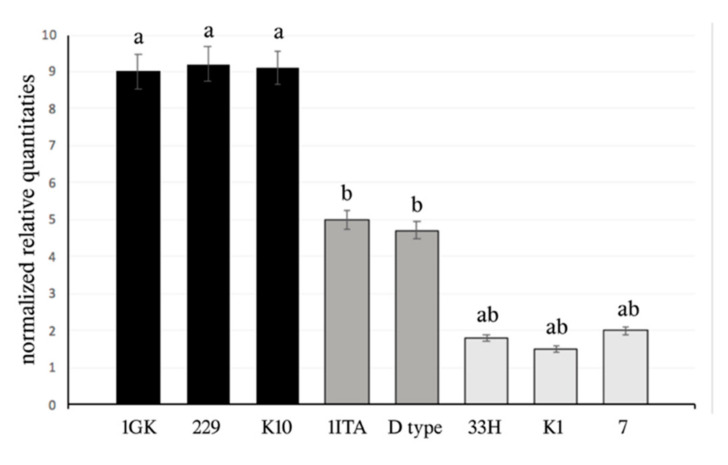
Normalized relative quantitative expression of for genes coding evidence high for expression level in clones 1GK, 229 and K10, whereas expression was scarce in the other clones and very low in clones 33H, K1 and 7, possessing severe mutations in the coding sequence. Bars indicate standard errors.

The analysis of the qPCR experiments (Figure 6) evidenced different expression levels of the *for* gene in the studied *M. persicae* clones. In particular, they can be subdivided into three different groups with high, medium and low *for* expression. Clones with severe mutations (33H, K1 and 7) all showed low *for* expression levels.

The higher *for* expression was observed in clones 1GK, 229 and K10, whose wingless adults and nymphs moved greater distances on crowded plants (exploratory behaviour) in respect to the other clones (Table 2).

These results are in agreement with data obtained in *D. melanogaster*, where the *rover* allele was isolated in larvae that traverse a large area while feeding [11]. Similar results have also been reported in *A. pisum*, where different behavioural variants have been observed in adults under crowded conditions, in addition to the typical production of winged morphs able to disperse over a long distance (Table 3). The behavioural differences observed in *A. pisum* have been related to the higher *for* expression in more mobile aphids [13]. As a consequence, the high *for* expression level observed in clones 1GK, 229 and K10 can be associated with the presence of exploratory behaviours due to crowded conditions.

Tares et al. [13] showed that *Apfor* is highly expressed in nymphs resulting in alate females, so they proposed an involvement of this gene in wing formation and in the flight capacity of the pea aphid. Similarly, clones 1GK, 229 and K10 simultaneously showed the greatest abundance of winged individuals and the highest *for* expression, suggesting the role of this gene in wing formation in the studied *M. persicae* clones. Moreover, the simultaneous presence of severe mutations and very low *for* expression was observed in clones 33H, K1 and 7 that lacked winged females on crowded plants, further supporting the role of the *for* gene in wing formation in *M. persicae*.

Similar results were observed in the honeybee *Apis mellifera*, where high *for* expression levels were related to the transition from nurses to foragers outside the hive [18,19], and in different ant species, where peaks in the *for* expression were observed in foragers [20,21,22]. These data, together with an extensive array of further studies describing *for* role [23], clearly support the suggestion that the *foraging* gene is an evolutionary conserved modifier of behaviour in insects.

Our data, as a whole, suggest that *for* alleles could allow for a differential adaptation of the *M. persicae* clones to unfavourable environmental conditions, such as overcrowding. In particular, the presence of short-term adaptive responses based on the presence of winged females and exploratory behaviours could confer a remarkable invasive potential to peach potato aphids, making them efficient pests.

Interestingly, some odours and pheromones enhance the expression of the *for* orthologue in nematodes [24]. This regulatory effect could also be an interesting further research topic in *M. persicae*, since alarm pheromone mediates the production of winged dispersal morphs in several aphid species [25]. The ability of alarm pheromone to regulate *for* expression could make some clones less prone to predation than other ones, improving their potential to become serious crop pests.

In the last decades, several studies analysed the expression of *for* in insects [23], but it is, at present, unclear if all individuals or only some of them differentially express this gene in the population. These data could be useful to establish a specific relationship between *for* gene expression and the behavioural plasticity of each population/species. The difficulty of these experiments is related to the presence of multiple unsynchronous individuals in insect populations. In laboratory conditions, as assessed by Nardelli et al. [26], it is possible to collect synchronous *M. persicae* individuals and populations so that it is easier to compare qPCR replicates, avoiding the high range of standard errors observed in previous experiments in other aphid species [13].

Lastly, we compared the *for* expression in *M. p. persicae* and *M. p. nicotianae* in order to confirm a previous hypothesis suggesting that differences in its expression were related to ecological specialization [14]. Our findings showed very different *for* expression in the two studied *M. p. nicotianae* clones (1GK and K1) so that our data did not support this proposal. Even if we agree that the adaptation to new host plants and/or the specialization for some of them may result from the simultaneous contribution of several genes, our data suggest that other mechanisms, such as the upregulation of detoxification-related genes [27] and gene duplications [28,29], could be at the basis of the ecological specialization observed in *M. p. nicotianae*.

Interestingly, the *for* gene has been suggested as a possible target for RNA interference-based field control strategies for the hemipteran *Bemisia tabaci* [30], further showing that the study of this gene may have an applicative interest for several agricultural pest insects.

## 4. Conclusions

The dispersal of winged females and the presence of exploratory behaviours represent key elements at the basis of the invasiveness of the *M. persicae* populations. As a consequence, the evaluation of the propensity of aphids to disperse in the fields, is essential to plan proper strategies for their control. In view of its involvement in the presence of both alate aphids and explorative behaviours, the screening of the *for* sequence could be useful as a tool for predicting invasions of cultivated areas by peach potato aphids.

## Figures and Tables

**Table 1:** Description of the main biological traits of the eight studied *M. persicae* clones. The column “Description” presents a general description of each clone and also distinguishes clones with generalist feeding traits as opposed to those specialized for tobacco, identified as *M. p. nicotianae*. The insecticide resistance is reported in term of resistance levels, with S being sensitive and R3 being the most resistant. Esterase confers resistance to insecticides such as organophosphates. Knockdown (kdr) and super knockdown (skdr) are involved in resistance to pyrethroids.

Clone	Year first Identification	Host	Description	Insecticide Resistance	Reproductive Mode
**1ITA**	2003	peach	susceptible to insecticides, generalist, common	S	holocyclic
**K1**	2004	potato	long-term resistant, identified as *M. p. nicotianae*, common	R3 resistance due to esterase gene duplication, kdr mutation	holocyclic
**K10**	2004	potato	long-term resistant, generalist, common	R3 resistance due to esterase gene duplication, kdr mutation	obligate parthenogenetic
**1GK**	2002	shepherd’s purse	susceptible, identified as *M. p. nicotianae*, common	S	holocyclic
**229**	2004	potato	long-term resistant, generalist, common	R3 resistance due to esterase gene duplication	holocyclic
**33H**	2012	potato	susceptible to insecticides, generalist, common	S	obligate parthenogenetic
**7**	2008	peach	susceptible to insecticides, generalist, common	S	obligate parthenogenetic
**D type**	2001	potato	long-term resistant, generalist, common	R3 resistance due to esterase gene duplication, kdr mutation, skdr mutation	obligate parthenogenetic

**Table 2:** Summary of results of the *for* nucleic sequence alignment in the studied *M. persicae* clones.

**1ITA**	**100%**							
**1GK**	99.91%	100%						
**229**	100%	99.91%	100%					
**K1**	99.82%	99.74%	99.82%	100%				
**D type**	100%	99.91%	100%	99.82%	100%			
**K10**	99.74%	99.65%	99.74%	99.56%	99.74%	100%		
**33H**	99.87%	99.78%	99.87%	99.69%	99.87%	99.61%	100%	
**7**	99.87%	99.78%	99.87%	99.69%	99.87%	99.61%	99.74%	100%
	**1ITA**	**1GK**	**229**	**K1**	**D type**	**K10**	**33H**	**7**

**Table 3:** Summary of the results observed in each *M. persicae* clone for the presence/absence of *for* mutations, alate females and exploratory behaviour.

Clone	Presence (+) or Absence (−) of Severe Mutations	Abundance (+) or Absence (−) of Alate Females	Presence (+) or Absence (−) of Explorative Behavior
1ITA	**−**	**+**	**−**
1GK	**−**	**++**	**+**
229	**−**	**++**	**+**
33H	**+**	**−**	**−**
K10	**−**	**++**	**+**
K1	**+**	**−**	**−**
7	**+**	**−**	**−**
D type	**−**	**+**	**−**
